# Behavioural Phenotyping of APP_swe_/PS1_δE9_ Mice: Age-Rrelated Changes and Effect of Long-Term Paroxetine Treatment

**DOI:** 10.1371/journal.pone.0165144

**Published:** 2016-11-04

**Authors:** Louise Ørum Olesen, Elena V. Bouzinova, Maurizio Severino, Mithula Sivasaravanaparan, Jørgen Bo Hasselstrøm, Bente Finsen, Ove Wiborg

**Affiliations:** 1 Department of Clinical Medicine, Health, Aarhus University, Risskov, Denmark; 2 Department of Neurobiology Research, Institute of Molecular Medicine, University of Southern Denmark, Odense C, Denmark; 3 Department of Forensic Medicine, Health, Aarhus University, Aarhus N, Denmark; Torrey Pines Institute for Molecular Studies, UNITED STATES

## Abstract

Alzheimer’s disease (AD) is a devastating illness characterized by a progressive loss of cognitive, social, and emotional functions, including memory impairments and more global cognitive deficits. Clinical-epidemiological evidence suggests that neuropsychiatric symptoms precede the onset of cognitive symptoms both in humans with early and late onset AD. The behavioural profile promoted by the AD pathology is believed to associate with degeneration of the serotonergic system. Using the APP_swe_/PS1_δE9_ model of AD-like pathology starting with 9 months old mice, we characterised long term non-cognitive behavioural changes measured at 9, 12, 15, and 18 months of age and applied principal component analysis on data obtained from open field, elevated plus maze, and social interaction tests. Long-term treatment with the selective serotonin reuptake inhibitor (SSRI) paroxetine was applied to assess the role of 5-HT on the behavioural profile; duration of treatment was 9 months, initiated when mice were 9 months of age. Treatment with paroxetine delays the decline in locomotion, in exploration and risk assessment behaviour, found in the APP/PS1 mice. APP/PS1 mice also exhibit low social activity and less aggressiveness, both of which are not affected by treatment with paroxetine. The APP/PS1 behavioural phenotype, demonstrated in this study, only begins to manifest itself from 12 months of age. Our results indicate that treatment with SSRI might ameliorate some of the behavioural deficits found in aged APP/PS1 mice.

## Introduction

Alzheimer’s disease (AD) is a devastating illness characterized by a progressive loss of cognitive, social and emotional functions, usually starting with memory impairment followed by more global cognitive deficits and neuropsychiatric symptoms, such as agitation, aggression, anxiety and depressive symptoms [1]. In terms of the neuropathology, one of the hallmarks of AD is the deposition of amyloid-ß (Aß) peptides in extracellular plaques, which starts years before the clinical symptoms [2]. Clinical-epidemiological evidence suggests that neuropsychiatric symptoms precede the onset of cognitive symptoms in both early and late onset AD [3, 4]. A dysfunction in the serotoninergic (5-HT) system has been implicated in both AD pathology [5, 6] and in the associated neuropsychiatric symptoms [7].

The Aß plaque burden has been shown to correlate to 5-HT4 receptor levels in AD [8], and post mortem studies have demonstrated reduced density of 5-HT neurons in the raphe nuclei in AD patients [5, 9]. This decrease in 5-HT neuron number correlates to reduced levels of 5-HT in various brain areas in patients suffering from AD (reviewed in [10, 11]), and the changes in the serotoninergic transmitter system might contribute to the occurrence of the neuropsychiatric symptoms observed in AD [7].

In the APP_swe_PS1_δE9_ transgenic mouse model of AD a progressive loss of forebrain serotoninergic axons has been demonstrated, from 12 months of age, to correlate with reduced levels of 5-HT [12]. In the same study a significant loss of serotoninergic neurons in the raphe nuclei in 18 months old mice was found to correlate with Aß deposits [12], which starts to develop from 3–4 months of age in this mouse model [13]. In the APP_swe_/PS1_δE9_ (APP/PS1) model Liu et al. [12] reports 5-HT neurodegeneration to precede the onset of anxiety-related behaviours, such as thigmotaxis, becoming evident from 18 months of age, and also to precede reduced motor activity in the open field (OF) test, apparent from 24 months of age. Most behavioural studies on APP/PS1 mice have been performed at a much earlier age, where 5-HT related dysfunction is not yet evident. Thus, to assess the possible effect of 5-HT related dysfunction on behaviour in the APP/PS1 model of AD, there is a need to assess behavioural changes well beyond 12 months of age, when loss of serotoninergic axons is initially observed.

Treatment with selective serotonin reuptake inhibitors (SSRIs) has been found to improve several cognitive and neuropsychiatric symptoms associated with AD [14]. Interestingly, the effect of antidepressant drugs has also been associated with lower Aß levels and plaque burden in both cortical and hippocampal regions in the APP/PS1 mice as well as in cortical regions of human subjects [15]. In fact, chronic treatment with citalopram, beginning at 3 months of age, before the onset of insoluble Aß deposits, was shown to impede the development of Aß deposition in 7-month-old APP/PS1 mice [15]. A recent study on 12-month old APP/PS1 mice, with established Aß pathology, indicated acute citalopram treatment to reduce Aß levels in brain interstitial fluid (ISF) in a dose-dependent manner, and chronic treatment to arrest individual plaque growth and to reduce new plaque formation in cortex [16]. Citalopram has also been found to reduce Aß levels in the CSF of healthy human subjects in one study [16], however, this finding was not replicated in a different study [17]. Long-term treatment with another SSRI, paroxetine, was found to delay development of Aß pathology and ameliorate behavioural deficits in another AD transgenic mouse model (3xTgAD) [18], which also exhibits dysfunctions in the 5-HT neurotransmitter system [19].

Here, we assessed the implications of a dysfunctional 5-HT system on mouse behaviour during aging using the APP/PS1 model of AD, starting at 9 months of age, when Aß pathology is well established [13, 20] and 5-HT axonal degeneration is not yet evident [21]. Hence, any abnormal behaviour at this time point will most likely not be attributed 5-HT signalling. Long-term treatment with paroxetine initiated at 9 months of age will further allow for evaluation of the correlation between development of AD-like pathology, 5-HT dysfunction, and behaviour. To our knowledge, the present study is the first longitudinal study investigating the effect of paroxetine on behavioural changes in the APP/PS1 mouse model. We describe the development of a behavioural phenotype from 9 to 18 months of age, which might be 5-HT-related. Furthermore, we analyse the effect of pharmacological intervention that is known to increase extracellular 5-HT levels in the brain [22]. We use principal component analysis to assess behavioural patterns that can be drawn from an extensive set of behavioural variables. We believe that this study will add to the overall behavioural characterisation of the APP/PS1 mouse model, as well as shed light upon the correlation between 5-HT dysfunction and abnormal behaviour.

## Materials and Methods

### Animals

#### Ethics statement

Mice were submitted to daily inspections and observations, however, no harmful effects of either genotype or treatment were observed and consequently no mice were euthanized before termination of the study. It is well-known, however, that hemizygous APPswe/PS1δE9 Tg mice show an increased mortality, without showing signs of previous disease, compared to littermate wildtype (WT) mice. Video−EEG monitoring shows that hemizygous Tg mice more frequently than wildtype mice, show seizure activity, which might be the reason behind the increased mortality. We observed an increased sudden (unexpected) mortality of Tg, but not WT mice treated with the initial high dose of paroxetine (30 mg/kg/day), while the survival of the Tg mice treated with the lower doses of paroxetine was unaffected. All casualties were reported to the ethics committee. Mainly coat state and body weight were used as readouts on a healthy state. At study termination mice were euthanized by a lethal dose of pentobarbital. Health status was monitored by daily inspections of coat state and general well being. Bodyweight was measured regularly. There was no apparent suffering of animals.

Double transgenic (Tg) APP_SWE_/PS_δE9_ (APP/PS1) mice, and littermate wild type (Wt) mice were bred on a B6C3 hybrid background (C57BL/6 X C3H/HeN) in the Biomedical Laboratory, University of Southern Denmark (n = 51) or at Taconic A/S, Denmark (n = 55). Mice were genotyped in-house, and animals carrying the rd/rd mutation [23, 24] were excluded. A total of 106 male mice were used in this study. Because of high levels of aggressive behaviour in this mouse model, animals were singly housed from 9 months of age. Animals were kept under standard laboratory conditions, *i*.*e*. food and water *ad libitum*, 12-h light/dark cycle (lights on at 6:00 p.m.) with constant temperature (21 ± 2°C) and humidity (52 ± 2%). Body weight and fluid intake was monitored regularly throughout the study. Animals were exposed to environmental enrichment consisting of wood splints bedding, chewing stick, access to bedding material, and mouse house. Cages were from Tecniplast, type III (425 x 266 x 155 mm). The Danish National Committee for Ethics in Animal Experimentation approved all animal procedures (2013-15-2934-00814).

### Paroxetine treatment

Paroxetine (Seroxat® oral solution 2 mg/ml, GSKline) was administered in drinking water initially at a dose of 30 mg/kg/day, which based on measurements of serum paroxetine was adjusted to 10 mg/kg/day and finally 5 mg/kg/day, corresponding to reported intraperitoneal dosing of 3xTgAD mice [18]. Treatment was initiated at 9 months of age, and was continued for a duration of 9 months. Tap water was used as vehicle. Serum paroxetine concentrations were monitored by UHPLC-MS/MS prior to, and subsequent to 3 months of treatment. Blood samples were collected and centrifuged for 10 min at 7000 rpm, the supernatant was stored at -80^°^C until further analysis.

The analysis of paroxetine was based on a previous method with few modifications [25]. Fifteen μl of serum was diluted with 45 μl water. Paroxetine-d4 was used as internal standard in a concentration of 300 nmol/l, and the mass spectrometer Agilent triple quadrupole 6460 (G6460B) was used for quantification. Two MRM transitions were monitored for both paroxetine and the internal standard (paroxetine: 330–192 and 330–70; paroxetine-d4: 334–196 and 334–74). Calibration was performed by linear calibration based on five-points. The measurement range was 50–20,000 nmol/l corresponding to the lower and upper limit of quantification. Effects of ion suppression were minimal. Precision was less than 15% coefficient of variation and trueness was within the range of 85–115%.

### Behavioural testing

Mice were subjected to a set of behavioural tests in order to assess the effect of age, genotype and treatment on various behavioural parameters. The Open Field (OF) and the Elevated Plus Maze (EPM) tests were used for evaluating anxiety-like behaviour, explorative behaviour and general locomotion. The Social Interaction (SI) test was used to evaluate social behaviour, including aggressive behaviour and memory for social interaction. Testing was done at 9, 12, 15 and 18 months of age, corresponding to 0, 3, 6 and 9 months of treatment, respectively. The animals were tested during the first half of the light phase in the light/dark cycle. Behavioural parameters were registered and scored by one or two experienced investigators. All test apparatuses were cleaned between individual test sessions with 70% ethanol and rinsed with water/wet paper towel.

Behavioural variables were abbreviated as indicated in Table 1. In the OF and EPM tests a total of 24 behavioural variables were scored and in the SI test 10 behavioural variables were registered.

**Table 1:** Abbreviation of behavioural variables scored during testing.

Test	Behavioural parameter	Abbreviation
Open Field	Total squares crossed	SqC_OF_
	Rearing against walls	RW_OF_
	Rearing, freestanding	R_OF_
	Central crossing	CC_OF_
	Immobility time	IT_OF_
	Stereotypy	St_OF_
	Grooming	Gr_OF_
	Faecal Boli	B_OF_
	Urine	U_OF_
	Freezing	Fr_OF_
Elevated Plus	Latency	Lat_EPM_
Maze	Immobility time	IT_EPM_
	Open arms, number of entries	OAN_EPM_
	Open arms, time spent in	OAT_EPM_
	Closed arms, number of entries	CAN_EPM_
	Closed arms, time spent in	CAT_EPM_
	Rearing, Closed arms	RC_EPM_
	Stretch-attend posture	SAP_EPM_
	Head dips, Open arms	HDO_EPM_
	Head dips, Closed arms	HDC_EPM_
	Grooming	Gr_EPM_
	Faecal Boli	B_EPM_
	Urine	U_EPM_
	Freezing	Fr_EPM_
Social Interaction,	Latency to first interaction	Lat_S1_
*Day 1*	Time spent in contact	T_S1_
	Contacts, number of	C_S1_
	Aggression, nb of incidents	Ag_S1_
	Aggression, time spent on	AgT_S1_
*Day 2*	Latency to first interaction	Lat_S2_
	Time spent in contact	T_S2_
	Contacts, number of	C_SI2_
	Aggression, nb of incidents	Ag_SI2_
	Aggression, time spent on	AgT_SI2_

After behavioural assessment at 9 months of age (treatment time = 0) animals were subsequently split into 4 experimental groups: Tg and Wt with no treatment, and Tg and Wt with paroxetine treatment. All together the study comprised 4 experimental groups, which were studied at 4 different time points. For each of the behavioural variables registered we made 198 observations in the OF and EPM tests and 221 observations in the SI test.

At 9 months of age, a total of 55 *(30 Wt; 25 Tg)* mice were included in the analysis. At 12 months of age a total of 46 mice *(12 TgVeh; 4 TgPrx; 15 WtVeh; 15 WtPrx)* mice were included in the analysis. At 15 months of age a total of 47 mice *(12 TgVeh; 6 TgPrx; 15 WtVeh; 15 WtPrx)* were included in the OF/EPM analysis, and 68 mice *(22 TgVeh; 9 TgPrx; 23 WtVeh; 14 WtPrx)* in the SI analysis. At 18 months of age a total of 50 mice *(12 TgVeh; 8 TgPrx; 14 WtVeh; 16 WtPrx)* were included in the OF/EPM analysis, and 68 mice *(12 TgVeh; 7 TgPrx; 19 WtVeh; 16 WtPrx)* in the SI analysis.

#### Open field test

The open field (OF) test was performed in a rectangular arena (60 cm × 80 cm, surrounded by 60 cm-high walls), made of dark painted wood. The arena was divided into 48 squares (10x10 cm) for visual scoring of activity. The mice were individually placed in one of the corners facing the perimeter, and allowed to explore the test arena freely for the entire test session (3 min). The following parameters were recorded; total distance travelled, rearing against the perimeter wall, free rearing, number of entries in the central area, time spent on immobility, stereotypy, grooming, and number of faecal boli as well as amount of urine traces and freezing incidents.

#### Elevated plus maze test

The Elevated Plus Maze (EPM) consisted of two sets of opposing arms, two open (30 cm × 5 cm) and two enclosed (30 cm × 5 cm, surrounded by 16 cm-high walls), made of black Plexiglas and elevated 80 cm above the floor. The mice were individually placed on the central platform, at the junction of the four arms, with head facing against an open arm, and left freely to explore the test arena for the entire test session (5 min). Following parameters were recorded: Latency to first entry into either of the arms, time of immobility, the number of entries into open and closed arms, the time spent in open and closed arms, rearing in open and closed arms, stretch-attend posture, head-dips, grooming and number of faecal boli, amount of urine traces, and freezing incidents.

#### Social interaction test

The social interaction (SI) test was performed on two consecutive days in a rectangular arena (60 cm × 80 cm, surrounded by 60 cm-high walls). The tests were on both days initiated by simultaneously placing a test mouse and a Wt male intruder mouse in opposite corners of the arena, facing the wall. The same test/intruder mice were paired on both days. During a 3-min test session the following parameters were registered; latency to initial contact, time spent in contact, number of social actions performed by the test mouse, number of aggressive incidents and time spent on aggressive behaviour. The intruder mice were age-matched male B6C3 (Wt) mice.

### 6E10 immunohistochemistry and Aß plaque load estimation

To estimate the Aß plaque load mice were deeply anaesthetized, and perfused with 10 ml phosphate buffered saline (PBS) followed by 20 ml of 4% paraformaldehyde (PFA). Brains were additionally fixed in 4% PFA for 24 h followed by 1% PFA for 24 h. The left hemisphere was marked and the brains were sectioned in a vibratome (Leica, DK) into 50 μm thick free-floating sections which were stored in de Olmos solution at -14°C.

Prior to staining sections were demasked in 70% formic acid diluted in H_2_O for 15 min at room temperature (RT), followed by rinsing in tris buffered saline (TBS) and TBS + 1% Triton (TBS/T), and pre-incubation in TBS/T + 10% fetal bovine serum (TBS/T/FBS). Then followed incubation with the biotinylated monoclonal mouse-anti-Aß antibody (6E10, SIG-39340, Biosite) diluted 1:500, first 30 min at RT and next o.n. at 4°C, using biotinylated isotype IgG1 diluted 1:50 (MG115, Invitrogen) as control. Next day the sections were rinsed in TBS/T, blocked in TBS/Methanol/H_2_0_2_ (8:1:1) for 10 min, and rinsed in TBS and TBS/T followed by incubation with Horse Radish Peroxidase-Streptavidin diluted 1:200 (RPN1231V, GE Healthcare) in TBS/T/FBS for 1 h at RT. After rinsing in TBS sections were developed with 3,3’-diaminobenzidine (DAB) for 10 min followed by rinsing in TBS. The sections were finally mounted on gelatine-coated glass slides, dehydrated in ethanol, cleared in xylene and coverslipped in Depex (Sigma).

The Aß plaque load was estimated in the right neocortex of two sections obtained from the level of the anterior commisure in 5 mice/group. For the estimation was used a stereological grid counting technique using the new CAST software (Visiopharm) as described in Babcock et al. (2015). The neocortex was delineated using a 4x magnification, and a grid stepping over the neocortex with a (x,y) step length of 286.5 μm x 216 μm and containing a similarly sized frame, with systematically placed crosses was used. Only plaques touching the centre of a cross were counted. The percentage of the neocortex covered by plaques was calculated as follows: Aß plaque load (%) = No. of Aß^+^ plaques touching a cross / No. of crosses in neocortex x 100%. The Aß plaque load in individual mice was calculated as the average of the Aß plaque load in the two sections. Data are presented as Mean ± SD.

## Data analysis

For all statistical analyses XLSTAT (version 2010.3.06) was used. All data sets were tested for normality of distribution by Shapiro-Wilk and Jarque-Bera tests. The Bartlett’s test was used to check the uniformity of variances. The criterion for statistical significance was set at p < 0.05. All data from OF, EPM and SI tests were subjected to principal component analysis (PCA). Aß plaque load was analysed by Student’s t-test. The Pearson product-moment correlation coefficient (Pearson’s r) analysis was used as a measure of correlation between Aß plaque load and behavioural variables.

### Principal component analysis

Principal component analysis (PCA) was applied in order to reduce the number of behavioural variables shown in Table 1. We conducted PCA on results from OF and EPM, examining general activity, exploratory activity and anxiety, and a separate PCA on data from SI, focusing on social behaviour. In both cases the Spearman’s correlation matrix was used. Data were subjected to Oblimin rotation with Kaiser normalization and Tau = 0, as recommended for behavioural data, to avoid bias towards the first principal component [26].

From principal component analysis on OF and EPM variables, 7 factors were extracted with eigenvalues higher than 1 (accounting for 65% of total variability). Examination of the Scree-plot indicated that a solution with 3 factors would be appropriate. As recommend by Abdi and Williams [27] we tried several rotation analyses on data from OF and EPM tests retaining different numbers of factors to assess robustness of the interpretation. Consequently, four principal components (PCs) were extracted, as they were considered of biological relevance.

The principal component analysis on results from the SI test extracted 4 factors with eigenvalue higher than 1 (accounting for 71% of total variability), which were retained for Oblimin rotation. Factor definitions and factor loadings obtained after Oblimin rotation are shown in Table 2 and Table 3, respectively.

**Table 2:** Description of factors and factor loadings obtained by PCA using Oblimin rotation on behavioural variables assessed by OF and EPM test.

Factors	Variables	Factor Loading^a^	Contribution (%) of the variables
Factor D1: Locomotion and anxiety-like behaviour	SqC_OF_	0.954	11.218
	RW_OF_	0.922	10.474
	R_OF_	0.940	10.895
	CC_OF_	0.958	11.318
	B_OF_	-0.960	11.365
	IT_OF_	-0.749	6.925
	St_OF_	-0.923	10.506
	Lat_EPM_	0.744	6.826
	B_EPM_	-0.957	11.299
Factor D2: Anxiety	OAN_EPM_	0.975	23.435
	OAT_EPM_	0.983	23.818
	CAT_EPM_	-0.831	17.011
	HDO_EPM_	0.913	20.529
Factor D3: Exploration and risk assessment	IT_EPM_	-0.851	14.622
	CAN_EPM_	1.008	20.514
	RC_EPM_	0.951	18.258
	HDC_EPM_	1.015	20.802
	SAP_EPM_	0.789	12.572
Factor D4: Stress-related vegetative behaviour	Gr_OF_	0.863	16.167
	U_OF_	-0.881	16.860
	Gr_EPM_	1.006	21.999
	U_EPM_	-0.920	18.374

^a^Factor loadings < 0.6 were not included in the table.

**Table 3:** Description of factors and factor loadings obtained by PCA using Oblimin rotation on behavioural variables assessed by SI test.

Factors	Variables	Factor loading^a^	Contribution (%) of the variables.
Factor S1:	Ag_S2_	1.001	43.863
Fighting/Aggression	AgT_S2_	0.991	42.996
Factor S2:	Lat_S1_	-1.021	33.731
Social activity	T_S1_	0.948	29.044
	C_S1_	0.691	15.430
	Lat_S2_	-0.811	21.286
Factor S3:		Ag_S1_	0.963	44.336
Aggression		AgT_S1_	1.015	49.224
Factor S4:	T_S2_	0.847	31.839
Memory for Social Interaction	C_S2_	0.976	42.249

^a^Factor loadings < 0.6 were not included in the table.

### Multivariate analysis of variance on principal components

The PCA scores were extracted and the resulting new variables (principal components) were normally distributed. Effect of age, genotype and treatment was tested using Multivariate Analysis of Variance followed by Fisher’s Least Significance Difference (LSD) *post hoc* test for pairwise analysis. The criterion for statistical significance was set at p < 0.05. Factor scores are presented as Mean ± SEM.

## Results

According to the study design animals at 9 months of age (treatment time = 0) were split into 4 experimental groups, which were studied at 4 different time points. Twenty-four variables were recorded in the OF and EPM tests, and 10 in the social interaction test. For each of the behavioural variables registered we made 198 observations in the OF and EPM tests and 221 observations in the SI test. Statistics on the individual behavioural variables from each of the 3 behavioural tests is available in supporting information. In order to reduce the number of behavioural variables from a total of 34 to 8, principal component analyses (PCA) were conducted. All behavioural variables described in Table 1 were included in the PCA, except for freezing behaviour, which was observed only on 3 occasions.

### Paroxetine levels

The serum paroxetine levels from mice at 9 and 12 months of age, corresponding to 0 and 3 months of treatment, are shown in Table 4.

**Table 4:** The serum paroxetine levels assessed by UHPLC-MS/MS.

Age (mth)	Vehicle		Paroxetine		
	Mean (nmol/l)	Mice (n)	Mean (nmol/l)	SEM	Mice (n)
9	n.d.	10	n.d.	n.d.	10
12	n.d.	10	103	16	16

n.d. = not detected (values <20 nmol/l).

### Body weight

Body weight data were collected throughout the study, as both age, and dosing with paroxetine can alter body mass. In this study there were no differences in weight measures among experimental groups, indicating no interference with the behavioural assessments.

### Principal component analysis of OF, EPM, and SI variables

Four types of behaviour, i.e. principal components (PCs), which have biological relevance in describing behaviour in the OF and EPM tests, were extracted by PCA. They were termed locomotion and anxiety-like behaviour, anxiety, exploration and risk assessment behaviour, and stress-related vegetative behaviour. The definition of the individual behavioural types (PCs) and the factor loadings obtained after Oblimin rotation are shown in Table 2. PCA on results from the SI test extracted 4 principal components with eigenvalue higher than 1, which were retained for Oblimin rotation. The four types of behaviour (PCs) were defined as aggression/fighting, social activity, aggression, and memory for social interaction. The definition of the individual behavioural types (PCs) and the factor loadings obtained after Oblimin rotation are shown in Table 3.

#### MANOVA on factorial scores obtained from the PCA

**Fig 1:**
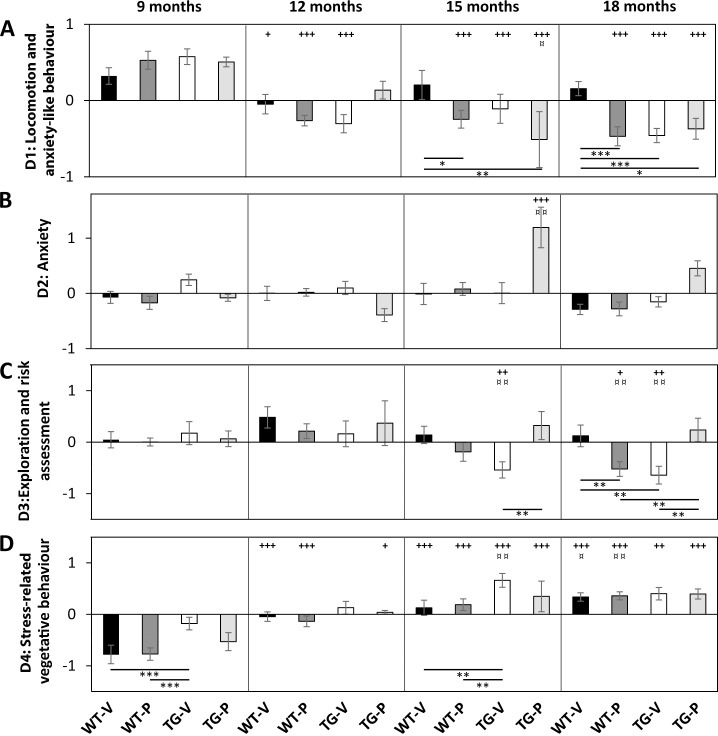
Effect of age, genotype and treatment on performance in OF (open field) and EPM (elevated plus maze) tests, analysed by principal component analysis. Factor scores for 4 types of behaviour (principal components) are shown in A-D. MANOVA was used to analyse significance in effect of age, genotype and treatment, followed by Fisher’s LSD *post hoc* test for group-wise comparisons: *, **, ***—p < 0.05, 0.01, 0.001 correspond to differences in factor scores between groups at 9, 12, 15 or 18 months of age; **+, ++, +++—**p < 0.05, 0.01, and 0.001 correspond to age-effect within groups compared to factor scores at 9 months; ¤, ¤¤, ¤¤¤—p < 0.05, 0.01, 0.001 correspond to intra-group comparisons of factor scores at 12 months. Bars indicate Means ± SEM Black bars, Wt (n = 14–15); white bars, Tg (n = 12–13); dark grey bars, Wt-paroxetine treated (n = 14–16); light grey bars, Tg-paroxetine treated (n = 6–12, and n = 4 at 12 months of age). 9, 12, 15 and 18 months of age correspond to 0, 3, 6 and 9 months of treatment, respectively.

**Fig 2:**
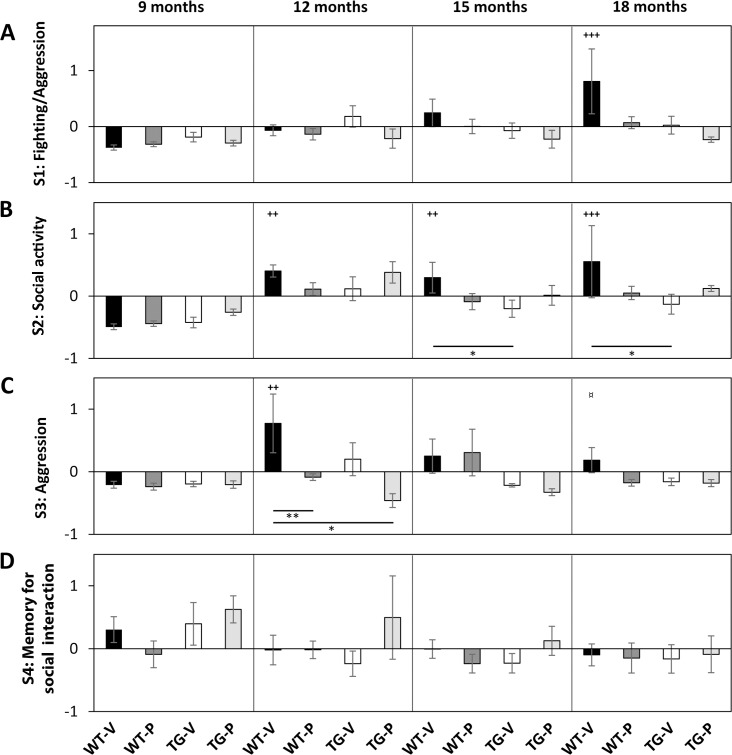
Effect of age, genotype and treatment on social interaction, tested on two consecutive days, analysed by principal component analysis. Factor scores for 4 types of behaviour (principal components) are shown in A-D. MANOVA was used to analyse significance in effect of age, genotype and treatment, following by Fisher’s LSD *post hoc* test for group-wise comparisons: *, **, ***—p < 0.05, 0.01, 0.001 correspond to differences in factor scores between groups at 9, 12, 15 or 18 months of age; +, ++, +++—p < 0.05, 0.01, and 0.001 correspond to age-effect within groups compared to factor scores at 9 months; ¤, ¤¤, ¤¤¤—p < 0.05, 0.01, 0.001 correspond to intra-group comparisons of factor scores at 12 months. Bars indicate Means ± SEM Black bars, Wt (n = 15–23); white bars, Tg (n = 12–22); dark grey bars, Wt-paroxetine treated (n = 14–16); light grey bars, Tg-paroxetine treated (n = 7–12, and n = 3 at 12 months of age). 9, 12, 15 and 18 months of age correspond to 0, 3, 6 and 9 months of treatment, respectively.

Overall MANOVA on factor scores derived from PCA demonstrates differences related to locomotion and anxiety-like behaviour, F(15,197) = 7.803, *p* < 0.0001; exploration and risk assessment behaviour, F(15,197) = 3.154, *p* = 0.00013; stress-related vegetative behaviour, F(15,197) = 11.024, *p* <0.0001; social activity (S2) F(15,220) = 2.560, *p* = 0.002; and aggression, F(15,220) = 1.864, *p* = 0.029. There are no significant differences in anxiety-like behaviour, F(15,197) = 1.689, *p* = 0.056; fighting/aggressive behaviour, F(15,220) = 1.594, *p* = 0.077, or memory for social interaction, F(15,220) = 1.329, *p* = 0.187. Factor scores (Mean ± SEM) along with results from pairwise comparison are shown in Fig 1 (OF and EPM tests) and Fig 2 (SI test).

### Behavioural phenotype at 9 months of age

Behavioural testing at 9 months of age was performed prior to treatment start. As such, the 4 experimental groups at 9 months of age, depicted in Figs 1 and 2, are pseudo groups used for monitoring the age-related changes in behaviour within the 4 experimental groups. To compare the behavioural phenotypes of Wt and Tg animals at 9 months of age (S1–S3 Tables), we used the average factor scores between the vehicle- and paroxetine-treated groups for Wt and Tg animals, respectively. We detected no changes in behaviour in 9 month-old Tg animals compared to Wt animals.

### Behavioural phenotype related to age, genotype and treatment

#### Age-related changes in behaviour analysed by PCA

Age-related changes in behaviour observed in the OF and EPM tests were related to locomotion, exploration and risk assessment behaviour, and stress-related vegetative behaviour with F(3,197) = 27.14 (p < 0.0001); 4.291 (p = 0.006) and 43.681 (*p* < 0.0001), respectively (S4 and S5 Tables). In the SI test age-related changes were observed only in social activity with F(3,220) = 6.643, p = 0.0002 (S6 Table). WtVeh animals demonstrated an increase in stress-related vegetative behaviour from 15 months of age (Fig 1D), in fighting/aggression (S1) at 18 months of age (Fig 2A), in social activity from 12 months of age (Fig 2B), and in aggression at 12 and 18 months of age (Fig 2C). TgVeh animals demonstrated reduced locomotion and anxiety-like behaviour during ageing (Fig 1A) from 12 months of age, reduced exploratory and risk assessments behaviour (Fig 1C) from 15 months of age, and increased stress-related vegetative behaviour from 15 months of age (Fig 1D). WtPrx animals demonstrated reduced locomotion and anxiety-like behaviour during ageing (Fig 1A) from 12 months of age, reduced exploratory and risk assessments behaviour (Fig 1C) from 18 months of age, and increased stress-related vegetative behaviour from 12 months of age (Fig 1D). TgPrx animals demonstrated reduced locomotion and anxiety-like behaviour (Fig 1A), and increased stress-related vegetative behaviour (Fig 1D) during ageing, both of which were evident from 15 months of age.

#### Changes in behaviour associated to the APP/PS1 genotype analysed by PCA

Changes associated to the genotype were observed in stress-related vegetative behaviour F(1,197) = 12.168, p = 0.001, and aggression F(1,220) = 5.545 (p = 0.019).

Multivariate analysis of variances facilitated investigation of the interactions of different parameters on the behaviour. We demonstrated an interaction between genotype and treatment in locomotion and anxiety-like behaviour F(1,197) = 3.919, p = 0.049, exploration and risk assessment behaviour F(1,197) = 15.216, p = 0.0001, and social activity F(1,220) = 4.851, p = 0.029. Interaction between age, genotype and treatment was observed in locomotion and anxiety-like behaviour F(3,197) = 3.039, p = 0.030, and in exploration and risk assessment behaviour F(3,197) = 3.617, p = 0.014.

At 18 months of age TgVeh demonstrated reduced locomotor and anxiety-like behaviour (Fig 1A), lower levels of exploratory behaviour (Fig 1C), and reduced social activity (Fig 2B) compared to WtVeh. The reduced level of social activity in the TgVeh animals was significant from 15 months of age. TgPrx animals show reduced locomotor activity from 15 months of age compared to WtVeh.

MANOVA on the aggression/fighting variable demonstrated no differences (p = 0.077) among groups, however, Fisher’s unprotected LSD test indicated reduced levels of aggression/fighting (Fig 2A) at 18 months of age in both TgVeh and TgPrx animals compared to WtVeh.

At 15 months of age TgVeh animals showed higher levels of stress-related vegetative behaviour (Fig 1D) compared to WtVeh. However, at 18 months of age the differences did not persist. We also observed higher levels of stress-related vegetative behaviour in TgVeh animals at 9 months of age compared to Wt animals.

#### Behavioural changes associated to long-term treatment with paroxetine

Long-term treatment with paroxetine was shown to have an effect on aggression (F(1,220) = 3.971, p = 0.048). MANOVA demonstrated an effect of the interaction between genotype and treatment on locomotion F(1,197) = 3.919, (p = 0.049), exploration and risk assessment behaviour F(1,197) = 14.542, (p = 0.0001), and social activity, F(1,220) = 4.851 (p = 0.029). An interaction between age and treatment F(3,197) = 3.248, (p = 0.023) was observed in locomotion and anxiety-like behaviour, and the interaction between age, genotype and treatment was found in both locomotion F(3,197) = 3.039, (p = 0.030), and exploration and risk assessment behaviour F(3,197) = 3.617, (p = 0.014).

#### TgVeh versus TgPrx

Long-term treatment with paroxetine was found to affect behaviour in the APP/PS1 mice. We observed higher levels of explorative and risk assessment behaviour from 15 months of age in TgPrx animals compared to TgVeh (Fig 1C). MANOVA revealed no age-related changes in anxiety-like behaviour (Fig 1B). However, Fisher’s unprotected LSD test indicated decreased levels of anxiety in TgPrx compared to WtVeh, significant from 15 months of age. There were no differences in anxiety-levels between TgVeh and TgPrx animals. In Tg animals social interaction (Fig 2) was not affected by long-term treatment with paroxetine. Although, there were indications of social activity (Fig 2B) being affected by treatment, as the reduced levels of social activity, observed in the TgVeh animals from 15 months of age compared to WtVeh, were not replicated in TgPrx animals.

#### WtVeh versus WtPrx

Long-term treatment with paroxetine also affected behaviour in the Wt animals. WtPrx animals have reduced locomotion and anxiety-like behaviour from 15 months of age (Fig 1A), reduced exploration and risk assessment behaviour at 18 months of age (Fig 1C), and reduced aggression/fighting behaviour at 18 months of age (Fisher’s Unprotected) (Fig 2A) compared to WtVeh.

### Aß-plaque load

To assess whether treatment with paroxetine affects Aß plaque load in the APP/PS1 mice, and whether Aß plaque load correlated to the behaviour observed in the OF test at 18 months of age, we obtained preliminary data on Aß plaque load from 5 TgVeh and 5 TgPrx mice. The Aß plaque load provides an estimate of the percentage of the neocortex that is occupied with 6E10+ Aß-containing plaques. We observed no effect of long-term treatment with paroxetine on Aß plaque load in neocortex of 18-month-old TgPrx mice (TgPrx: 15.9% ± 4.2% vs. TgVeh: 15.0% ± 3.0%, n = 5/group, p = 0.709), nor did we find any correlation between Aß plaque load in the neocortex and behaviour in the OF test (Total number of squares crossed) in neither untreated (**r** = 0.016; p = 0.841), nor treated (**r** = 0.052; p = 0.713) mice.

## Discussion

Alzheimer’s disease is a neurodegenerative disorder associated with Aß deposition, synaptic and neuronal loss [9, 28], and altered serotoninergic neurotransmission [29–31]. The APP/PS1 transgenic (Tg) mouse model was developed to mimic AD associated amyloid pathology in humans based on mutations in APP (amyloid precursor protein) and PS1 (presenilin1) genes, resulting in the formation of Aß plaques [32]. In the present study we have used a number of behavioural tests to characterise behavioural abnormalities that can be attributed to either ageing and/or associated with formation of Aß plaques.

### Possible role of serotonin in AD-like disease in mice

Liu et al. (12) have demonstrated 5-HT neuronal degeneration in APP/PS1 mice, associated with lower levels of 5-HT and inversely to increasing Aß plaque load. Treatment with citalopram has been shown to delay or interfere with the development of Aß plaques [15, 16]. The use of another SSRI, paroxetine, in the 3xTgAD mouse model, also exhibiting reduced 5-HT levels [19], has likewise shown to delay or interfere with the development of Aß pathology and to ameliorate behavioural deficits [18]. We examined whether long-term treatment with paroxetine normalised behavioural changes observed in Tg mice by using paroxetine to increase 5-HT levels. Our results demonstrated that Tg mice develop a distinct phenotype, evident from 15 to 18 months of age. We also demonstrated that long-term treatment with paroxetine affects behaviour in the Tg mice. Moreover, our study design allowed us to investigate the side effects of paroxetine on behaviour in Wt mice.

AD has been associated with non-cognitive neuropsychological symptoms related to changes in 5-HT levels, such as activity disturbances, affective disturbances, aggression, stereotypic behaviour, and anxiety [11, 33]. Behavioural symptoms in the APP/PS1 mouse model, which are correlated to neuropsychological clinical symptoms, include disturbances in locomotor activity [34], anxiety [35, 36], aggression, and social behaviour [37–39]. The present study thus focuses on performance in OF and EPM tests, in which animals are allowed to explore the environment undisturbed, and where different behavioural parameters can be assessed, and on the SI test, where interest to engage into social interaction is analysed. All three tests can be used to evaluate the aforementioned behavioural symptoms.

### Describing behavioural patterns using principal component analysis

Generalising our findings, 4 principal components (PCs) describing behaviour in the OF and the EPM and 4 PCs describing social behaviour in the SI test were extracted by principal component analyses (PCAs). The behaviour described by the PC is directly associated with variables from the original data set that have a positive factor loading, and inversely associated with variables, which have a negative factor loading. The PCs from the analysis on OF and EPM variables were defined as locomotion and anxiety-like behaviour, anxiety, exploration and risk assessment, and stress-related vegetative behaviour. Locomotion (PC D1) is directly associated with variables related to motor activity (such as total squares crossed, and rearing against walls, which for mice can be included as yet another measure of general locomotion, and inversely associated with variables such as immobility time and stereotypy in OF, which is interpreted as anxiety-related behaviour. A higher factor score on this variable indicates higher levels of locomotor and anxiety-like activity. Anxiety-like behaviour (PC D2) is directly associated with variables having a high anxiolytic valence, and is inversely associated with time spent in the closed arms of the EPM. A high factor score on this variable indicates reduced anxiety-like behaviour. Exploration and risk assessment (PC D3) is described by variables that are related to explorative behaviour and Stretch-Attend-Postures, all observed within the closed arms of the EPM. Immobility time in EPM has a negative factor load on PC D3. A high factor score on this variable indicates a high level of explorative and risk assessment behaviour. Stress-related vegetative behaviour (PC D4) is directly associated with grooming actions and inversely associated with the amount of urine traces. Some authors correlate grooming and urination with anxiety-like behaviour [40], but in our study these two behavioural variables registered in both OF and EPM tests were always describing a separate PC when performing rotation analyses, indicating that they describe a type of behaviour, which is not directly correlated to anxiety-related behaviour. We consider this type of behaviour as stress-related vegetative behaviour, and a high factor score on this variable indicates a high level of stress-related vegetative behaviour.

Principal components from the analysis on social interaction variables (Table 3) differentiate mainly according to the test day in question, thus number of aggressive incidents and time spent in aggression on the second day define PC S1 (aggression/fighting). Latency to initial contact on both days and social interaction on day 1 define PC S2 (social activity). The number of aggressive incidents and the time spent in aggression on day 1 define PC S3 (aggression). The total time of contact and the number of contacts on day 2 define PC S4 (memory for social interaction). Both the S2 and the S4 behavioural variables might be a measure of anxiety-like behaviour, since an increase in the time spent engaging in social contact is indicative of reduced anxiety-like behaviour [41–43]. For all 4 PCs, a high factor score indicates more activity of the specific type of behaviour in question.

### No behavioural changes in 9 months old APP/PS1 mice compared to Wt

At 9 months of age there is no behavioural performance deficits in the Tg mice compared to Wt littermate mice. Although a difference in stress-related vegetative behaviour between Wt and TgVeh animals at 9 months of age was observed when the mice were split into experimental groups, no differences were observed when the analysis comprised all Tg and Wt mice. Any biological variation in stress-related vegetative behaviour at 9 months of age in the Tg group is overruled by age-related changes, seen as there are no differences among groups at 18 months of age.

There are some reports indicating increased locomotion in the APP/PS1 mouse model [44], and reduced anxiety-like behaviour at 7 months of age [35, 36]. Consistent with our findings, however, there are also studies reporting no changes in OF activity in APP/PS1 mice at 7 months of age [36], at 10–15 months of age [45], and no changes in anxiety-related behaviour at 10–15 months of age [45]. At 9 months of age 5-HT axonal degeneration is not yet evident, and 5-HT levels are still unchanged according to Liu et al. [12]. Hence, any behavioural changes in the APP/PS1 mouse model at this age are most likely not caused by changes in 5-HT levels.

### Development of the APP/PS1 phenotype

Normal ageing progression as analysed in this study is characterised by an increase in stress-related vegetative behaviour and increased social activity, and no changes in locomotion, anxiety, exploration, aggression, and memory for social interaction. There are indications of an age-related increase in aggression/fighting in Wt animals, but the importance of this behaviour might be overestimated, as the differences between groups are demonstrated without correction for multiple comparisons. These behavioural changes, demonstrated in the Wt group, are due to normal ageing processes and are unrelated to genotype and long-term treatment with paroxetine.

Ageing in Tg mice is associated with other parameters than in Wt animals, and we demonstrate reduced locomotion and anxiety-like behaviour, evident from 12 months of age, reduced social activity at 15 months of age, and reduced explorative behaviour from 15 months of age. Also, Tg animals do not demonstrate increased fighting behaviour at 18 months of age as observed in Wt animals.

It is well known, that AD patients exhibit changes in social behaviour [33], such as increased aggressiveness, and reluctance to engage in social contact. Other murine models of AD also display increased aggressive behaviour [46, 47]. In the present study, changes in aggression levels do not characterise the AD-like pathology, and are negligible compared to Wt.

Our data demonstrate reduced social activity in Tg mice, which is in line with findings by Filali et al. [39], who report that APP_swe_/PS1_A246E_ mice are less willing to engage in social contact and show reduced social memory. We find no changes in the memory for social interaction (S4). We do report an increase in aggressive behaviour in Wt mice on the first day of the SI test compared to the second day. This is not reproduced in Tg mice, inferring that the ability to recall social interaction is found in Wt mice alone.

Most of the commonly used AD mouse models exhibit increased locomotor activity (reviewed by Webster et al. [48]). However, no differences in overall activity or even hypoactivity has also been reported in APP/PS1 mice [49, 50]. Our study supports the findings of reduced locomotion in APP/PS1 mice.

According to Liu et al. [12] anxiety, measured by increased thigmotaxis and reduced exploration in the OF test, is not evident until 18 and 24 months of age, respectively. In line with these findings, we see no differences in anxiety levels measured by the EPM test at 18 months of age. However, Tg mice have reduced social activity in the SI test from 15 months of age, which might be indicative of reduced anxiety-like behaviour. There are also reports indicating that APP/PS1 mice exhibit decreased levels of anxiety, as assessed by EPM [35, 36, 49], while others fail to replicate the anxiety-like phenotype [45, 51]. The test design of the EPM apparatuses might have contributed to the divergent findings. In the latter of the reported study, the EPM apparatus used was 5 cm wide [51], the same as in the present study. This might have introduced a high anxiogenic factor compared to the 10 cm wide apparatus used by Lalonde et al. [36, 49], resulting in divergent results. The presence of anxiety-related behaviour in the APP/PS1 mouse model is thus widely discussed [35, 36, 52] and our findings support the findings of unchanged anxiety-levels, at least up to 18 months of age.

### SSRI-dependent changes in behaviour

Long-term treatment with paroxetine affects the behavioural phenotype in the APP/PS1 mice on some but not all behavioural parameters, suggesting that not all behavioural deficits in the Tg mice can be ascribed to changes in/reduced 5-HT levels. Paroxetine-treatment does not induce behavioural changes in either locomotor or anxiety-like activity, stress-related vegetative, aggressive or social behaviour in Tg animals. However explorative and risk assessment behaviour is affected, and from 15 months of age after 6 months of paroxetine-treatment, behaviour in Tg animals is reverted to a normal Wt phenotype. Paroxetine-induced reduction in anxiety-levels is implied from 15 months of age in Tg animals, however, at 18 months of age the effect is decreased. The power of the statistical analysis might be improved by increasing the number of observations, but an anxiolytic effect of paroxetine treatment is inferred at this point. Neither Wt nor Tg mice exhibit changes in anxiety-related behaviour with age and paroxetine only affects this behaviour in Tg mice. Paroxetine-treatment also affects social behaviour in the Tg mice. Untreated Tg mice display reduced levels of social activity from 15 months of age, which is not replicated in the paroxetine treated Tg animals.

### Effect of paroxetine on Aß plaque load

We observed no difference in neocortical Aß plaque load after 9 months treatment with paroxetine. Although data are still preliminary this observation is potentially important since 4 months of treatment with Citalopram was shown to impede the development of Aß pathology in 7-month-old APP/PS1 mice [15] and 5 months of treatment with paroxetine reduced Aß pathology and ameliorated behavioural deficits in 10-month-old 3xTgAD mice [18]. Our finding of no difference in neocortical Aß plaque load may partly explain why we see only partial effects of the long-term treatment with paroxetine on behaviour. We also investigated for correlations between Aß plaque load and behaviour in the OF test (Total number of squares crossed), as we found the locomotor activity to be affected in Tg animals. There was no correlation between Aβ plaque load and ‘Total number of squares crossed’ in neither untreated or treated mice. This indicates that the neocortical Aß plaque load *per se is* not responsible for the changes in locomotor activity seen in the Tg mice.

### Side effects of treatment with paroxetine

To control for side effects of paroxetine on behaviour, the WtPrx group was included in the study, and the results demonstrate that long-term treatment with paroxetine has distinct effects on Wt and Tg animals. In Wt mice the negative side effects of treatment are pronounced, and the paroxetine-treated Wt mice display a behavioural phenotype similar to untreated Tg mice, characterised by a decline in both motor activity, and explorative and risk assessment behaviour as well as in social behaviour. This differential effect on behaviour in Wt and Tg mice emphasises the importance of applying a disease model when examining the effect and mechanisms of drug action.

### Longitudinal study

Most behavioural studies performed on the APP/PS1 mouse model have assessed behaviour at a certain age, providing a snapshot of the behavioural phenotype. Longitudinal studies are few, and the evaluation of results from studies at different ages, and with different methodologies is difficult. We believe that for a detailed description of the behavioural changes, longitudinal studies are needed to assess the age-related progression of the APP/PS1 phenotype. Our results, based on a large number of animals, indicate that the APP/PS1 non-cognitive behavioural phenotype is only beginning to emerge from 15 months of age.

We took advantage of PCA to provide general conclusions on the behavioural phenotype of APP/PS1 mice. PCA allowed us to mathematically combine different variables to pinpoint major behavioural patterns. This procedure is an important part of the study and the importance of the general conclusions derived from the results of the PCA is highly valuable, as the conclusions are based on the combined results obtained from the data sets of the whole study.

## Conclusion

The APP/PS1 mouse model is appropriate for investigating the progression of an AD-like pathology. The PCA is a helpful tool in distinguishing specific types of behaviour and to generalise results derived from a big data set.

We find that APP/PS1 mice exhibit a specific age-related behavioural phenotype that manifests itself from about 15 months of age when 5-HT levels are reduced [21].

Long-term effect of chronic treatment with the SSRI, paroxetine, in APP/PS1 mice is significant regarding behaviour related to anxiety, exploration and risk assessment, and activity. This indicates that the analysed behavioural changes are related to changes in serotonin levels. Our results indicate that the effect of paroxetine can be evaluated by locomotion, exploration and risk assessment as well as anxiety-related behaviour. The evaluation of changes in social behaviour is relevant only in aged APP/PS1 mice. Longitudinal studies are necessary to describe the development of a behavioural phenotype resembling the neuropsychiatric symptoms seen in AD, as well as the effect of treatment with SSRI.

## Supporting Information

S1 Table Results of open field test obtained from APP_swe_PS1_δE9_ and WT mice at the age of 9 months before the initiation of the treatment compared by KWH test. (DOCX)

S2 Table Results of elevated plus maze test obtained from APP_swe_PS1_δE9_ and WT mice at the age of 9 months before the initiation of the treatment compared by KWH test. (DOCX)

S3 Table Results of social interaction tests performed in two consequent days and obtained from APP_swe_PS1_δE9_ and WT mice at the age of 9 months before the initiation of the treatment and compared by KWH test. (DOCX)

S4 Table Results of open field test obtained from APP_swe_PS1_δE9_ and WT mice at the age of 9, 12, 15, and 18 months compared by KWH test and Dunn’s group-wise analysis. (DOCX)

S5 Table Results of elevated plus maze test obtained from APP_swe_PS1_δE9_ and WT mice at the age of 9, 12, 15, and 18 months compared by KWH test and Dunn’s group-wise analysis. (DOCX)

S6 Table Results of social interaction tests on 2 consecutive days obtained from APP_swe_PS1_δE9_ and WT mice at the age of 9, 12, 15, and 18 months compared by KWH test and Dunn’s group-wise analysis. (DOCX)
