# Supplementary material for: Behavioural Phenotyping of APPswe/PS1δE9 Mice: Age-Rrelated Changes and Effect of Long-Term Paroxetine Treatment
Source: PLoS One. 2016 Nov 4;11(11):e0165144. doi: 10.1371/journal.pone.0165144 (PMC5096719; doi:10.1371/journal.pone.0165144)
Supplement: S1 Table — (DOCX) [file pone.0165144.s001.docx]

## S1 Table

Results of open field test obtained from **APP_swe_PS1_dE9_** and WT mice at the age of 9 months before the initiation of the treatment compared by KWH test

| **Open Field** | | | | | |
| --- | --- | --- | --- | --- | --- |
| **Variable** | **Age (mth)** | **WT** | **TG** | ***K_(3.841)_*** | ***P*** |
| TDist-OF | 9 | 121.67±52.80 | 151.60±56.86 | 3.472 | Ns |
| RW-OF | 9 | 11.73±6.91 | 14.20±8.15 | 1.251 | Ns |
| R-OF | 9 | 2.33±3.14 | 1.16±1.99 | 3.414 | Ns |
| CC-OF | 9 | 4.80±2.51 | 6.80±3.61 | 4.630 | 0.031 |
| Gr-OF | 9 | 0.53±0.68 | 0.92±1.26 | 1.074 | Ns |
| U-OF | 9 | 2.33±2.45 | 1.72±2.19 | 1.154 | Ns |
| B-OF | 9 | 0.80±1.13 | 1.24±1.39 | 1.431 | Ns |
| IT-OF | 9 | 0.40±0.93 | 0.20±0.50 | 0.495 | Ns |
| St-OF | 9 | 0.07±0.25 | 0.08±0.28 | 0.035 | Ns |
| Fr-OF | 9 | 0.10±0.31 | 0.00±0.00 | 2.596 | Ns |
| SqCOF - Total squares crossed; RWOF - Rearing against walls; ROF – Rearing (free standing); CCOF - Central crossing; ITOF - Immobility time; StOF – Stereotypy; GrOF – Grooming; BOF - Faecal Boli; UOF – Urine; FrOF - Freezing | | | | | |
