# Supplementary material for: Behavioural Phenotyping of APPswe/PS1δE9 Mice: Age-Rrelated Changes and Effect of Long-Term Paroxetine Treatment
Source: PLoS One. 2016 Nov 4;11(11):e0165144. doi: 10.1371/journal.pone.0165144 (PMC5096719; doi:10.1371/journal.pone.0165144)
Supplement: S2 Table — (DOCX) [file pone.0165144.s002.docx]

## S2 Table

Results of elevated plus maze test obtained from **APP_swe_PS1_dE9_** and WT mice at the age of 9 months before the initiation of the treatment compared by KWH test

| **Elevated Plus Maze** | | | | |  |
| --- | --- | --- | --- | --- | --- |
| **Variable** | **Age (mth)** | **WT** | **TG** | ***K_(3.841)_*** | ***P*** |
| Lat-EPM | 9 | 6.83±8.94 | 15.13±32.88 | 0.398 | Ns |
| IT-EPM | 9 | 27.75±27.65 | 31.35±29.80 | 0.312 | Ns |
| FrN-EPM | 9 | 0.29±1.28 | 0.04±0.20 | 0.093 | Ns |
| OAN-EPM | 9 | 0.21±0.48 | 0.21±0.41 | 0.006 | Ns |
| OAT-EPM | 9 | 2.86±11.18 | 9.57±28.10 | 0.328 | Ns |
| CAN-EPM | 9 | 2.21±1.45 | 2.37±2.02 | 0.038 | Ns |
| CAT-EPM | 9 | 0.07±0.25 | 290.43±28.10 | 0.328 | Ns |
| RC-EPM | 9 | 6.11±3.29 | 6.05±4.36 | 0.007 | Ns |
| HDO-EPM | 9 | 0.07±0.37 | 0.80±1.98 | 2.439 | Ns |
| HDC-EPM | 9 | 3.61±3.10 | 5.31±4.53 | 1.671 | Ns |
| Gr-EPM | 9 | 0.82±1.15 | 2.31±2.44 | 5.907 | 0.015 |
| SAP-EPM | 9 | 5.25±2.93 | 6.02±4.10 | 0.365 | Ns |
| U-EPM | 9 | 0.96±1.07 | 0.33±0.62 | 7.037 | 0.008 |
| B-EPM | 9 | 1.32±1.29 | 1.37±1.18 | 0.059 | Ns |
| LatEPM – Latency; ITEPM - Immobility time; OANEPM - Number of entries in Open arms; OATEPM – Time spent in Open arms; CANEPM – Number of entries in Closed arms; CATEPM – Time spent in Closed arms; RCEPM – Rearing in Closed arms; SAPEPM - Stretch-attend posture; HDOEPM - Head dips in Open arms; HDCEPM - Head dips in Closed arms; GrEPM – Grooming; BEPM – Number of Faecal Boli; UEPM – Number of Urine traces; FrEPM – Number of Freezing actions | | | | | |
