# Supplementary material for: Behavioural Phenotyping of APPswe/PS1δE9 Mice: Age-Rrelated Changes and Effect of Long-Term Paroxetine Treatment
Source: PLoS One. 2016 Nov 4;11(11):e0165144. doi: 10.1371/journal.pone.0165144 (PMC5096719; doi:10.1371/journal.pone.0165144)
Supplement: S3 Table — (DOCX) [file pone.0165144.s003.docx]

## S3 Table

Results of social interaction tests performed in two consequent days and obtained from **APP_swe_PS1_dE9_** and WT mice at the age of 9 months before the initiation of the treatment and compared by KWH test

| **Social Interaction** | |  |  |  |  |
| --- | --- | --- | --- | --- | --- |
| **Variable** | **Age (mth)** | **WT** | **TG** | ***K_(3.841)_*** | ***P*** |
| LatS1 | 9 | 73.07±55.48 | 69.20±53.58 | 0.000 | ns |
| TS1 | 9 | 29.47±20.99 | 35.44±20.70 | 0.447 | ns |
| CS1 | 9 | 8.40±6.09 | 8.84±5.42 | 0.012 | ns |
| AgS1 | 9 | 0.07±0.37 | 0.12±0.44 | 0.533 | ns |
| AgTS1 | 9 | 0.10±0.55 | 0.08±0.28 | 0.502 | ns |
| LatS2 | 9 | 50.03±41.65 | 45.92±48.29 | 0.632 | ns |
| TS2 | 9 | 25.23±18.37 | 42.28±34.74 | 4.909 | 0.027 |
| CS2 | 9 | 7.03±5.01 | 8.20±4.64 | 1.409 | ns |

LatS1 - Latency to first interaction on Day1; TS1 - Time spent in contact on Day1; CS1 – Number of Contacts on Day1; AgS1 – Number of Aggression actions on Day1; AgTS1 – Time spent in Aggression on Day1; LatS2 - Latency to first interaction on Day2; TS2 - Time spent in contact on Day2; CSI2 – Number of Contacts on Day2; AgSI2 – Number of Aggression actions on Day2; AgTSI2 – Time spent in Aggression on Day2
