# Supplementary material for: Behavioural Phenotyping of APPswe/PS1δE9 Mice: Age-Rrelated Changes and Effect of Long-Term Paroxetine Treatment
Source: PLoS One. 2016 Nov 4;11(11):e0165144. doi: 10.1371/journal.pone.0165144 (PMC5096719; doi:10.1371/journal.pone.0165144)
Supplement: S4 Table — (DOCX) [file pone.0165144.s004.docx]

## S4 Table

Results of open field test obtained from **APP_swe_PS1_dE9_** and WT mice at the age of 9, 12, 15, and 18 months compared by KWH test and Dunn’s group-wise analysis

| **Open Field** | | | | | | | |
| --- | --- | --- | --- | --- | --- | --- | --- |
| **Variable** | **Age (mth)** | **WTveh** | **WTprx** | **TGveh** | **TGprx** | ***K_(24.996)_*** | ***P*** |
| Total Distance in squares (TDist-OF) | 9 | 106.47±46.14 | 136.87±56.12 | 152.46±74.31 | 150.67±32.05 | 56.616 | <0.0001 |
|  | 12 | 87.20±58.78 | 77.20±44.43 **^a^** | 69.42±47.69 **^a^** | 155.25±34.88 **^x.y.z^** |  |  |
|  | 15 | 170.00±81.87**^a.b^** | 110.07±64.65 **^x^** | 101.58±65.60 **^x^** | 74.50±63.76 |  |  |
|  | 18 | 127.14±37.19 | 80.44±65.63 **^a.x^** | 48.00±29.76 **^a.c.x^** | 95.13±75.36 |  |  |
| Rearing against walls  (RW-OF) | 9 | 11.40±7.07 | 12.07±6.98 | 14.08±10.56 | 14.33±4.81 | 72.999 | <0.0001 |
|  | 12 | 4.67±4.43**^a^** | 4.93±4.06 **^a^** | 5.33±4.66 **^a^** | 3.25±2.87 **^a^** |  |  |
|  | 15 | 10.40±5.84**^b^** | 5.00±3.88 **^a.x^** | 5.17±4.15 **^a.x^** | 4.33±7.76 **^a.x^** |  |  |
|  | 18 | 5.57±4.05**^a.c^** | 2.88±3.32 **^a^** | 3.00±2.86 **^a^** | 3.50±2.00 **^a^** |  |  |
| Rearing, free standing  (R-OF) | 9 | 1.93±3.28 | 2.73±3.06 | 1.46±2.50 | 0.83±1.27 | 41.647 | 0.0003 |
|  | 12 | 2.40±3.62 | 0.87±0.99 | 0.33±0.65 **^x^** | 0.25±0.50 |  |  |
|  | 15 | 2.73±3.86 | 0.93±1.27 **^a^** | 0.25±0.62 **^x^** | 0.33±0.52 |  |  |
|  | 18 | 2.71±2.49 | 0.56±1.36 **^a.x^** | 0.17±0.39 **^x^** | 0.38±0.52 **^x^** |  |  |
| Central Crossings   (CC-OF) | 9 | 4.47±2.70 | 5.13±2.36 | 5.92±4.05 | 7.75±2.93 | 74.599 | <0.0001 |
|  | 12 | 2.13±2.33**^a^** | 1.13±1.92 **^a^** | 1.33±1.44 **^a^** | 6.75±3.86 **^x.y.z^** |  |  |
|  | 15 | 3.20±2.37 | 1.93±2.37 **^a^** | 2.50±2.58 **^a^** | 1.17±1.60 **^a.b^** |  |  |
|  | 18 | 2.43±2.06 | 1.63±2.28 **^a^** | 1.33±1.37 **^a^** | 2.13±2.10 **^a.b^** |  |  |
| Immobility time (sec)   (IT-OF) | 9 | 0.47±1.06 | 0.33±0.82 | 0.31±0.63 | 0.08±0.29 | 92.011 | <0.0001 |
|  | 12 | 0.47±1.06 | 0.33±0.82 | 0.00±0.00 | 0.00±0.00 |  |  |
|  | 15 | 18.40±37.82 | 29.79±34.53 **^a.b^** | 34.42±47.79 **^a.b^** | 25.00±23.38 **^a.b.x^** |  |  |
|  | 18 | 16.57±22.96**^a.b^** | 53.25±48.32 **^a.b^** | 57.42±41.13 **^a.b.c^** | 22.00±26.05 **^a.b^** |  |  |
| Stereotypy  (St-OF) | 9 | 0.07±0.26 | 0.07±0.26 | 0.15±0.38 | 0.00±0.00 | 34.512 | 0.003 |
|  | 12 | 0.00±0.00 | 0.13±0.35 | 0.17±0.39 | 0.00±0.00 |  |  |
|  | 15 | 0.07±0.26 | 0.21±0.43 | 0.08±0.29 | 1.50±2.07 **^a.b.x.z^** |  |  |
|  | 18 | 0.07±0.27 | 0.38±0.50 **^a.x^** | 0.42±0.51 **^c.x^** | 0.50±0.53 **^a.b.x^** |  |  |
| Grooming  (Gr-OF) | 9 | 0.47±0.64 | 0.60±0.74 | 1.08±1.61 | 0.75±0.75 | 14.030 | n.s. |
|  | 12 | 1.00±1.36 | 1.00±1.81 | 1.25±1.76 | 0.25±0.50 |  |  |
|  | 15 | 0.80±1.26 | 0.71±0.91 | 2.83±3.54 | 1.50±1.76 |  |  |
|  | 18 | 1.79±1.76**^a^** | 1.19±2.43 **^x^** | 1.58±1.78 | 0.50±0.76 |  |  |
| Boli  (B-OF) | 9 | 1.07±1.39 | 0.53±0.74 | 1.15±1.52 | 1.33±1.30 | 9.137 | n.s. |
|  | 12 | 1.47±1.77 | 1.40±1.40 | 1.33±1.23 | 0.75±0.50 |  |  |
|  | 15 | 1.47±2.17 | 1.43±1.79 | 1.50±1.73 | 1.33±0.82 |  |  |
|  | 18 | 0.79±1.19 | 1.44±1.31 | 1.17±0.94 | 1.75±1.49 |  |  |
| Urine  (U-OF) | 9 | 2.53±2.95 | 2.13±1.92 | 1.15±1.41 | 2.33±2.74 | 40.631 | 0.0004 |
|  | 12 | 0.67±1.05 **^a^** | 0.27±0.59 **^a^** | 0.42±0.67 | 0.00±0.00 **^a^** |  |  |
|  | 15 | 0.93±1.33 | 0.43±1.34 **^a^** | 0.58±2.02 | 0.67±0.82 |  |  |
|  | 18 | 1.00±1.47 | 0.31±0.79 **^a^** | 0.67±1.78 | 0.13±0.35 **^a^** |  |  |
| Freezing   (Fr-OF) | 9 | 0.07±0.26 | 0.13±0.35 | 0.00±0.00 | 0.00±0.00 | 16.840 | n.s. |
|  | 12 | 0.00±0.00 | 0.00±0.00 | 0.00±0.00 | 0.00±0.00 |  |  |
|  | 15 | 0.00±0.00 | 0.00±0.00 | 0.00±0.00 | 0.00±0.00 |  |  |
|  | 18 | 0.00±0.00 | 0.00±0.00 | 1.33±4.62 | 0.00±0.00 |  |  |

**^a^** vs. 9 months; **^b^** vs. 12 months; **^c^** vs. 15 months; **^x^** vs. WTveh; **^y^** vs. WTprx; **^z^** vs. TGveh for *P*<0.05 by Dunn’s post KWH test; ^n.s.^ No significant differences by KWH test
