# Supplementary material for: Behavioural Phenotyping of APPswe/PS1δE9 Mice: Age-Rrelated Changes and Effect of Long-Term Paroxetine Treatment
Source: PLoS One. 2016 Nov 4;11(11):e0165144. doi: 10.1371/journal.pone.0165144 (PMC5096719; doi:10.1371/journal.pone.0165144)
Supplement: S5 Table — (DOCX) [file pone.0165144.s005.docx]

## S5 Table

Results of elevated plus maze test obtained from **APP_swe_PS1_dE9_** and WT mice at the age of 9, 12, 15, and 18 months compared by KWH test and Dunn’s group-wise analysis

| **Elevated Plus Maze** | | | | | | | | |
| --- | --- | --- | --- | --- | --- | --- | --- | --- |
| **Variable** | **Age (mth)** | **WTveh** | | **WTprx** | **TGveh** | **TGprx** | **F_(15.167)_** | ***P*** |
| Latency (sec)  (LAT-EPM) | 9 | | 5.27±7.18 | 8.40±10.44 | 22.17±44.27 | 7.50±9.93 | 20.324 | n.s. |
|  | 12 | | 9.13±10.25 | 4.80±5.68 | 7.50±9.47 | 3.00±0.82 |  |  |
|  | 15 | | 5.20±3.95 | 9.14±15.62 | 10.75±9.43 | 7.67±16.38 |  |  |
|  | 18 | | 2.07±2.30 | 3.86±4.13 | 3.00±4.20 | 1.40±1.47 |  |  |
| Immobility time (sec)  (IT-EPM) | 9 | | 36.21±33.69 | 19.29±17.17 | 26.75±21.46 | 36.33±37.20 | 77.815 | <0.0001 |
|  | 12 | | 52.73±36.18 | 70.53±51.40 **^a^** | 100.50±67.91 **^a^** | 62.75±40.84 |  |  |
|  | 15 | | 68.80±44.62 | 92.86±67.59 **^a^** | 129.25±69.76 **^a^** | 41.33±50.46 **^z^** |  |  |
|  | 18 | | 88.36±64.65 **^a^** | 135.36±64.34 **^a.b^** | 147.00±52.00 **^a.x^** | 28.20±38.20 **^x.y.z^** |  |  |
| Entries into open arms  (OAN-EPM) | 9 | | 0.21±0.56 | 0.21±0.41 | 0.33±0.47 | 0.08±0.29 | 21.249 | n.s. |
|  | 12 | | 0.13±0.35 | 0.13±0.35 | 0.08±0.29 | 0.00±0.00 |  |  |
|  | 15 | | 0.20±0.41 | 0.07±0.27 | 0.00±0.00 | 0.33±0.52 |  |  |
|  | 18 | | 0.00±0.00 | 0.07±0.25 | 0.08±0.29 | 0.20±0.34 |  |  |
| Time (sec) spent in open arms   (OAT-EPM | 9 | | 5.36±15.64 | 0.36±0.89 | 9.17±21.87 | 10.00±34.64 | 21.545 | n.s. |
|  | 12 | | 2.87±8.72 | 7.53±19.95 | 3.75±12.99 | 0.00±0.00 |  |  |
|  | 15 | | 3.80±12.85 | 14.29±53.45 | 0.00±0.00 | 24.50±46.88 |  |  |
|  | 18 | | 0.00±0.00 | 0.36±1.24 | 0.83±2.89 | 5.60±9.47 |  |  |
| Entries into closed arms  (CAN-EPM) | 9 | | 2.36±1.63 | 2.07±1.28 | 2.17±1.77 | 2.58±2.31 | 38.767 | 0.001 |
|  | 12 | | 5.40±3.09 **^a^** | 3.00±2.27 **^x^** | 2.42±2.43 **^x^** | 3.25±1.26 |  |  |
|  | 15 | | 4.67±3.92 | 4.00±2.96 | 1.42±0.67 **^x.y^** | 4.67±4.41 **^z^** |  |  |
|  | 18 | | 5.64±4.13 **^a^** | 3.64±3.07 | 1.83±1.59 **^x^** | 4.00±2.51 **^z^** |  |  |
| Time (sec) spent in closed arms   (CAT-EPM) | 9 | | 294.64±15.64 | 299.64±0.89 | 290.83±21.87 | 290.00±34.64 | 75.080 | <0.0001 |
|  | 12 | | 275.53±28.57 **^a^** | 283.33±38.94 **^a^** | 242.33±86.97 **^a^** | 291.00±5.72 **^a^** |  |  |
|  | 15 | | 274.40±39.91 **^a^** | 266.07±71.87 **^a^** | 256.25±47.93 **^a^** | 246.83±42.08 **^a^** |  |  |
|  | 18 | | 293.93±8.35 **^b.c^** | 292.36±8.88 **^a^** | 287.92±20.32 | 234.60±43.03 **^a.x.y.z^** |  |  |
| Rearing in Closed arms  (RC-EPM) | 9 | | 7.14±3.93 | 5.07±2.19 | 7.17±4.98 | 4.83±3.35 | 29.430 | 0.014 |
|  | 12 | | 4.87±4.00 | 7.67±5.91 | 7.33±6.95 | 5.75±6.60 |  |  |
|  | 15 | | 5.60±4.29 | 4.07±2.95 | 2.83±5.10 **^a.b.x^** | 6.33±7.45 |  |  |
|  | 18 | | 6.43±4.29 | 4.07±3.13 | 2.00±2.73 **^a.b.x^** | 4.80±4.70 |  |  |
| Stretch-attend postures   (SAP-EPM) | 9 | | 4.21±2.76 | 6.29±2.81 | 6.58±5.12 | 5.42±2.71 | 79.546 | <0.0001 |
|  | 12 | | 5.93±2.31 | 7.47±3.87 | 6.75±4.97 | 7.00±6.16 |  |  |
|  | 15 | | 3.47±2.13 **^b^** | 2.50±2.50 **^a.b^** | 2.33±2.84 **^a.b^** | 5.33±2.88 **^y.z^** |  |  |
|  | 18 | | 1.57±1.60 **^a.b.c^** | 1.14±0.88 **^a.b^** | 2.92±2.47 **^a.b^** | 2.00±0.53 **^a^** |  |  |
| Head dips in Open arms  (HDO-EPM) | 9 | | 0.14±0.52 | 0.00±0.00 | 0.92±1.71 | 0.67±2.31 | 35.694 | 0.002 |
|  | 12 | | 1.07±1.44 | 1.53±1.88 **^a^** | 1.42±1.68 | 0.00±0.00 **^z^** |  |  |
|  | 15 | | 1.13±1.68 | 0.64±1.45 | 1.83±3.66 | 5.83±9.68 |  |  |
|  | 18 | | 0.14±0.53 | 0.21±0.40 | 0.67±1.30 | 2.40±1.74 **^a.b.x.y.z^** |  |  |
| Head dips in Closed arms   (HDC-EPM) | 9 | | 4.21±3.63 | 3.00±2.45 | 5.75±4.67 | 4.83±4.53 | 19.721 | n.s. |
|  | 12 | | 6.53±6.79 | 6.87±3.98 | 6.42±7.03 | 10.25±6.95 |  |  |
|  | 15 | | 4.33±2.64 | 4.64±3.86 | 3.42±2.71 | 4.33±3.98 |  |  |
|  | 18 | | 6.07±5.46 | 2.50±2.77 | 4.17±3.56 | 7.40±8.31 |  |  |
| Grooming   (GR-EPM) | 9 | | 0.57±0.73 | 1.07±1.44 | 2.83±2.85 | 1.75±1.86 | 28.244 | 0.02 |
|  | 12 | | 1.67±1.84 **^a^** | 0.87±1.19 | 1.83±1.95 | 1.75±1.71 |  |  |
|  | 15 | | 3.60±5.32 **^a^** | 2.36±2.62 | 4.50±4.25 | 5.17±5.19 |  |  |
|  | 18 | | 2.57±2.50 **^a^** | 3.21±2.37 **^a.b^** | 3.92±4.42 | 3.60±2.86 |  |  |
| Boli  (B-EPM) | 9 | | 1.29±1.33 | 1.36±1.29 | 1.25±1.23 | 1.50±1.17 | 29.661 | 0.013 |
|  | 12 | | 2.13±1.85 | 2.60±1.06 **^a^** | 3.00±2.00 **^a^** | 1.75±1.26 |  |  |
|  | 15 | | 1.80±1.90 | 2.50±1.65 | 1.92±1.93 | 1.33±1.51 |  |  |
|  | 18 | | 0.71±1.27 **^b^** | 2.36±1.44 **^x^** | 1.50±1.68 **^b^** | 1.40±1.01 |  |  |
| Urine  (U-EPM) | 9 | | 0.93±1.33 | 1.00±0.76 | 0.17±0.55 **^x.y^** | 0.50±0.67 **^y^** | 60.052 | <0.0001 |
|  | 12 | | 0.40±0.74 | 0.27±0.59 **^a^** | 0.08±0.29 | 0.00±0.00 |  |  |
|  | 15 | | 0.13±0.35 **^a^** | 0.07±0.27 **^a^** | 0.00±0.00 | 0.17±0.41 |  |  |
|  | 18 | | 0.07±0.27 **^a^** | 0.00±0.00 **^a^** | 0.00±0.00 | 0.00±0.00 **^a^** |  |  |
| Freezing  (Fr-EPM) | 9 | | 0.00±0.00 | 0.57±1.80 **^x^** | 0.08±0.28 **^x^** | 0.00±0.00 **^y^** | 26.847 | 0.03 |
|  | 12 | | 0.00±0.00 | 0.00±0.00 **^a^** | 0.00±0.00 **^a^** | 0.00±0.00 |  |  |
|  | 15 | | 0.07±0.26 | 0.00±0.00 **^a^** | 0.00±0.00 **^a^** | 0.00±0.00 |  |  |
|  | 18 | | 0.00±0.00 | 0.00±0.00 **^a^** | 0.00±0.00 **^a^** | 0.00±0.00 |  |  |

**^a^** vs. 9 months; **^b^** vs. 12 months; **^c^** vs. 15 months; **^x^** vs. WTveh; **^y^** vs. WTprx; **^z^** vs. TGveh for *P*<0.05 by Dunns’s post KWH test; **^n.s.^** No significant differences by KWH test
