# Supplementary material for: Behavioural Phenotyping of APPswe/PS1δE9 Mice: Age-Rrelated Changes and Effect of Long-Term Paroxetine Treatment
Source: PLoS One. 2016 Nov 4;11(11):e0165144. doi: 10.1371/journal.pone.0165144 (PMC5096719; doi:10.1371/journal.pone.0165144)
Supplement: S6 Table — (DOCX) [file pone.0165144.s006.docx]

## S6 Table

Results of social interaction tests on 2 consecutive days obtained from **APP_swe_PS1_dE9_** and WT mice at the age of 9, 12, 15, and 18 months compared by KWH test and Dunn’s group-wise analysis

| **Social Interaction test** | | | | | | | |
| --- | --- | --- | --- | --- | --- | --- | --- |
| **Variable** | **Age**  **(mth)** | **WTveh** | **WTprx** | **TGveh** | **TGprx** | **K_(24.996)_** | ***P*** |
| Latency (sec) to first contact on Day1 (S1) | 9 | 80.20±53.34 | 65.93±58.49 | 72.46±53.56 | 65.67±55.74 | 46.694 | <0.0001 |
|  | 12 | 26.20±23.16 **^a^** | 23.29±19.31 **^a^** | 38.92±46.16 | 19.00±10.54 |  |  |
|  | 15 | 21.29±19.00 **^a^** | 44.86±43.86 | 41.14±48.00 **^a^** | 28.33±15.10 |  |  |
|  | 18 | 16.89±15.65 **^a^** | 45.31±43.58 **^x^** | 49.67±58.39 | 25.57±16.96 |  |  |
| Time (sec) spent in contact on Day1 (S1) | 9 | 31.40±17.36 | 27.53±24.56 | 33.38±17.19 | 37.67±24.53 | 32.102 | 0.006 |
|  | 12 | 50.33±28.25 | 32.36±12.92 | 44.58±20.38 | 34.00±17.58 |  |  |
|  | 15 | 38.33±16.21 | 33.64±19.81 | 31.00±23.57 **^b^** | 29.78±10.33 |  |  |
|  | 18 | 51.74±17.76 **^a.c^** | 48.69±23.71 **^a.b.c^** | 40.08±22.17 | 33.71±19.17 **^x^** |  |  |
| Number of Contacts on Day1 (S1) | 9 | 8.93±5.60 | 7.87±6.71 | 7.77±4.34 | 10.00±6.38 | 16.916 | n.s. |
|  | 12 | 8.13±6.59 | 7.29±2.55 | 6.92±3.65 | 17.00±7.55 |  |  |
|  | 15 | 9.25±4.19 | 7.29±5.17 | 6.90±4.16 | 11.11±5.16 |  |  |
|  | 18 | 9.00±5.26 | 7.63±6.17 | 6.92±5.38 | 7.71±5.96 |  |  |
| Number of Aggression actions on Day1 (S1) | 9 | 0.13±0.52 | 0.00±0.00 | 0.00±0.00 | 0.25±0.62 | 44.656 | <0.0001 |
|  | 12 | 3.53±6.15 **^a^** | 0.36±1.08 **^x^** | 1.67±3.98 **^a^** | 0.00±0.00 **^x^** |  |  |
|  | 15 | 1.50±4.45 **^b^** | 0.14±0.36 | 0.00±0.00 **^b^** | 0.00±0.00 |  |  |
|  | 18 | 2.53±5.88 **^b^** | 0.25±0.77 | 0.25±0.87 **^b^** | 0.00±0.00 |  |  |
| Time (sec) spent in Aggression on Day1 (S1) | 9 | 0.20±0.77 | 0.00±0.00 | 0.00±0.00 | 0.17±0.39 | 32.250 | 0.001 |
|  | 12 | 4.87±10.25 **^a^** | 0.29±0.83 | 1.42±3.23 **^a^** | 0.00±0.00 |  |  |
|  | 15 | 2.83±8.25 | 5.64±16.76 **^a^** | 0.00±0.00 **^b.x.y^** | 0.00±0.00 **^y^** |  |  |
|  | 18 | 0.00±0.00 **^b.c^** | 0.00±0.00 **^c^** | 0.00±0.00 **^b^** | 0.00±0.00 |  |  |
| Latency (sec) to first contact on Day2 (S2) | 9 | 49.00±36.42 | 51.07±47.58 | 45.85±51.78 | 46.00±46.50 | 29.990 | 0.012 |
|  | 12 | 18.67±17.40 **^a^** | 22.43±22.08 | 31.92±33.39 | 41.67±26.69 |  |  |
|  | 15 | 19.92±21.10 **^a^** | 33.21±36.22 | 47.95±48.44 **^x^** | 46.33±38.32 |  |  |
|  | 18 | 17.05±13.36 **^a^** | 49.19±60.45 | 47.17±53.29 **^x^** | 25.57±11.96 |  |  |
| Time (sec) spent in contact on Day2 (S2) | 9 | 25.80±15.53 | 24.67±21.39 | 44.54±45.11 | 39.83±20.00 | 16.139 | n.s. |
|  | 12 | 37.00±18.19 | 38.43±23.01 | 36.00±19.90 | 35.33±20.55 |  |  |
|  | 15 | 35.75±24.09 | 31.93±15.36 | 32.24±23.56 | 30.33±13.82 |  |  |
|  | 18 | 43.89±26.47 | 43.00±29.00 | 37.50±15.87 | 34.71±18.80 |  |  |
| Number of Contacts on Day2 (S2) | 9 | 8.27±5.50 | 5.80±4.30 | 7.00±4.43 | 9.50±4.68 **^y^** | 32.673 | 0.005 |
|  | 12 | 5.33±5.75 **^a^** | 5.86±2.74 | 3.83±2.79 | 8.67±5.86 |  |  |
|  | 15 | 5.83±3.51 | 4.00±2.60 | 4.86±4.04 | 7.67±4.24 **^y^** |  |  |
|  | 18 | 4.16±4.39 **^a^** | 3.69±4.00 **^b^** | 4.33±3.80 | 5.57±4.50 |  |  |
| Number of Aggression actions on Day2 (S2) | 9 | 0.00±0.00 | 0.00±0.00 | 0.00±0.00 | 0.00±0.00 | 28.275 | 0.02 |
|  | 12 | 1.13±2.39 | 0.64±1.91 | 2.17±3.51 **^a^** | 0.67±1.15 |  |  |
|  | 15 | 2.25±5.44 **^a^** | 0.57±1.87 | 0.86±2.87 | 0.33±1.00 |  |  |
|  | 18 | 5.05±13.22 **^a^** | 0.50±1.75 | 0.58±1.73 | 0.14±0.38 |  |  |
| Time (sec) spent in Aggression on Day2 (S2) | 9 | 0.00±0.00 | 0.00±0.00 | 0.00±0.00 | 0.00±0.00 | 28.931 | 0.016 |
|  | 12 | 1.00±2.24 | 0.79±2.67 | 2.92±5.82 **^a^** | 1.33±2.31 |  |  |
|  | 15 | 6.17±13.83 **^a^** | 4.43±6.57 **^a.b^** | 1.52±5.49 | 1.67±5.00 |  |  |
|  | 18 | 9.00±22.19 **^a^** | 2.13±5.02 | 2.58±6.83 **^a^** | 0.14±0.38 |  |  |

**^a^** vs. 9 months; **^b^** vs. 12 months; **^c^** vs. 15 months; **^x^** vs. WTveh; **^y^** vs. WTprx; **^z^** vs. TGveh for *P*<0.05 by Dunn’s post KWH test;

**^n.s.^** No significant differences by KWH test
